# Portion Size: Latest Developments and Interventions

**DOI:** 10.1007/s13679-017-0239-x

**Published:** 2017-03-06

**Authors:** Ingrid Steenhuis, Maartje Poelman

**Affiliations:** 1 Department of Health Sciences, Faculty of Earth & Life Sciences, Vrije Universiteit Amsterdam, Amsterdam Public Health Research Institute, Amsterdam, The Netherlands; 2 Department of Human Geography and Spatial Planning, Utrecht University, Heidelberglaan 2, PO Box 80115, 3508 TC Utrecht, The Netherlands

**Keywords:** Portion size, Portion-size interventions, Energy intake, Portion-size effect

## Abstract

**Purpose of Review:**

The aim of this review is to provide an overview of (1) underlying mechanisms of the effect of portion size on energy intake, (2) external factors explaining the portion size effect and (3) interventions and measurements aimed at food portion size.

**Recent Findings:**

Previous studies have shown that portion sizes have increased in recent decades. Many experimental studies have been conducted to unravel the mechanisms underlying the portion-size effect on food intake (e.g. the appropriateness mechanism, the ‘unit bias’ mechanism, the ‘previous experience/expectation’ mechanism, the ‘visual cue’ mechanism and the ‘bite size’ mechanism). In addition, external factors have been found to drive food portion selection and consumption (e.g. value for money, mindless eating, levels of awareness, estimation bias. Research on several interventions (ranging from ‘providing information’ to ‘eliminating choice’) have been conducted, but remain scarce, especially intervention studies in which portion size is a key focus in weight loss. Moreover, only three new instruments with respect to portion control behavior have been developed.

**Summary:**

There is considerable evidence for the portion-size effect on energy intake. However, the work on interventions targeting portion size and measurements for portion control behavior are limited. Moreover, from the literature it is not yet clear what type of interventions work best, for whom and in what context.

## Introduction

In the past few decades, the prevalence of obesity and overweight in adults has increased, more than doubling since the 1980s [1]. Overweight and obesity are related to various health problems such as diabetes mellitus type 2, cardiovascular disease and several types of cancer [2]. Moreover, overweight and obesity are associated with certain psychosocial problems, such as depression and anxiety disorders [3]. In tandem with the increased prevalence of overweight and obesity, an increase in food portion sizes has also been observed [4]. These growing food portion sizes might well be one of the factors contributing to the increased prevalence of overweight and obesity in adults.

In recent years, several studies have been conducted to shed light on the mechanisms behind the portion-size effect. Moreover, increasing attention has been paid to interventions aimed at portion size. This paper presents an overview of recent developments. Notwithstanding the fact that the portion-size effect is also present in children, this paper focuses on the adult population, as the effects are largest in adults [5•, 6•].

## Trends in Food Portion Sizes

Very few studies have been conducted into trends in food portion sizes, with most carried out in or before 2009. Studies on developments in food portion sizes have been conducted in the USA [4, 7–10], the UK [11, 12], Denmark [13] and the Netherlands [14]. All of these studies showed that portion sizes of numerous energy-dense foods have increased in the past decades, also highlighting the introduction of ‘super-sized’ portions. Examining changes in food portions between 2000 and 2009, Young and Nestle [10] showed that this trend has continued into the present century, noting the introduction of many new larger sized portions [10]. With respect to home-cooked meals, Wansink and Payne [15] have demonstrated that portion sizes, as stated in a popular cookbook, have increased over the past 70 years [15], while Eidner et al. [16] found the same trend over the past 100 years in the case of a Danish cookbook [16]. Given this apparent continuing and constant change in portion sizes and noticeable international differences [17], it is important to follow up these studies and continue the monitoring of food portion sizes globally.

## Effects of Portion Size on Energy Intake

Numerous studies have demonstrated that people’s energy intake increases when they are offered larger portions. In a meta-analytic review, Zlatevska et al. [6•] showed that doubling a food portion leads, on average, to an increase in energy consumption of 35% [6•]. In their Cochrane review, Hollands et al. [5•] also found a consistent effect of portion size on energy intake. They estimated that the energy intake from food and non-alcoholic beverages attributable to differences in product sizes was between 215 and 279 kcal/day [5•]. Benton [18] emphasized the fact that we need more studies of real-life situations to establish the true effect of portion size on energy intake in daily life [18]. Such a study was conducted by French et al. [19], who demonstrated that exposure to a high-energy lunch over a period of 6 months led to significant increases in energy intake and weight gain in a real-life work setting [19].

## Underlying Mechanisms of the Portion-Size Effect

Many experimental studies have been conducted over the past decade to unravel the mechanisms underlying the portion-size effect on food intake. Dual process theory may be important to the understanding of these mechanisms, distinguishing between a system of deliberate, conscious reasoning and a more automatic and fast system of associative reasoning [20]. Some studies have attempted to unravel the deliberate processes that steer portion-size selection using a cognitive approach to explain the portion-size effect whereas other studies have attempted to unravel the non-deliberate processes using an automatic approach in explaining the portion-size effect [21, 22•]. Below, we briefly summarize the key mechanisms that are frequently highlighted when explaining the portion-size effect. A more in-depth and comprehensive explanation of the underlying mechanisms of the portion-size effect can be found in recently published papers by Herman et al. [23] and English et al. [22•, 23].

- *The appropriateness mechanism*. Although the evidence is not conclusive and there have been mixed results over recent years, the most prevalent explanation for the portion-size effect is the concept of ‘appropriateness’. According to this perspective, a food portion sets a norm and guides the amount consumed. Consequently, the portion size (rather than hunger or satiety) directs food consumption and steers food intake. Marchiori et al. used the term ‘anchoring effect’(aligning it with the appropriateness effect) to explain how portion sizes work as an anchor or reference point [24]. As Herman et al. [23] mention in their review, the ‘fractional version’ of the appropriateness mechanism should be further explored, as people often do not consume the entire portion, but only a ‘fraction’ of the portion size served [23]. Questions remain about what fraction of the portion is appropriate, does this differ for different people and how does this amount emerge?
- *The ‘unit bias’ mechanism*. This model suggest people see one serving (e.g. one sandwich, one can of food, one biscuit) as appropriate to consume at once, irrespective of its size [25]. Kerameas et al. [26] argued that the term ‘segmentation bias’ might be more applicable, as they found that people eat less when food is divided into smaller units [26]. Eating a number of smaller units rather than eating one larger unit is perceived by consumers as more impulsive and less appropriate [27].
- *The* ‘*previous experience*/*expectation*’ *mechanism*. Previous experiences may steer portion-size selection. For example, previous experience of the ‘degree of fullness produced by a food’ impacts on the portion size selected and consumed at a later point in time [28]. A study by Brunstrom et al. [29] showed that cognitive expectations about satiety and satiation influence the portion size selected [29].
- *The* ‘*visual cue*’ *mechanism*. The portion-size effect might be partly explained by visual cues, for example dishware size. People may use visual cues to steer their portion-size intake. For example, the degree of ‘plate emptiness’ may activate meal termination [21]. The Delboeuf illusion is also frequently mentioned in the literature to explain how similar portion sizes of food appear larger served on a small plate than on a large plate, and this steers individuals to judge portions differently [30].
  The size of dishware has been studied more intensively in recent years. This is of interest as it has been found that people frequently use large-sized dishware at home [31]. However, with respect to plate size, a recent systematic review and meta-analysis showed that there is no consistent effect of plate size on food intake [32]. It has also been found that plate size does not affect people’s estimation of the portion size [33]. However, a modelling study showed a positive association between plate size and energy available [34]. Moreover, the rim width of the plate might also influence portion-size selection (the larger the rim width, the less is served) [35]. In buffet experiments, smaller plates did not result in less food being consumed [36, 37]. Libotte et al. [38] demonstrated that participants increased their vegetable serving when using the large-sized plate [38].
- *The bite size mechanism*. Similarly to the use of laundry powder, toothpaste or spaghetti—where people pour out or use more when the package size is larger [39]—it has been found that people increase their bite size when food portions are larger [21, 40].

## External Factors Explaining the Portion-Size Effect

Additional factors that are not addressed by the portion-size effect—or the way the portion is served—may also impact on the portion size consumed. A wide range of external factors influence food consumption (and surplus portion-size intake), but a few important external factors that are frequently suggested in the literature as affecting the portion size selected and consumed are explained below.

- *Value for money*. Larger portion sizes can usually be offered at a proportionally low cost, since the cost of the food itself is relatively low compared to other costs such as labour [10]. The concept of ‘value for money’ has been identified as an important incentive in relation to consumers *selecting* larger portions [39, 41]. Consumers are likely to pay marginally more for a larger portion when several food portion options are available because they feel they are getting more value for money. Value for money can therefore be seen as a mechanism underlying the consumption of larger portions.
- *Mindless eating*. In the past decades, many studies have been conducted showing that individuals who engage in mindless eating (eating while distracted and not focused on the food they are consuming) consume larger amounts. Mindless eating impairs an individual’s ability to accurately estimate the amount of food they consume and they are hindered from making deliberate decisions on how much they should eat [42]. When eating mindlessly, individuals report lower degrees of fullness and a greater desire to eat compared to those who are not distracted [43]. Consequently, individuals are at risk of consuming surplus amounts when sufficient food is available [44]. Watching television, playing a computer game, listening to the radio and dining with others are factors that typically lead to mindlessly eating larger amounts than intended [45–48]. A recent review by Robinson et al. indicated that mindless eating is associated with a moderate increase in the immediate intake as well as a larger intake at a later point in time. Moreover, the effect of mindless eating was independent of dietary restraint [32].
- *Awareness and estimation bias*. People have difficulties in estimating amounts of food [49] and, moreover, are unaware of reference portion sizes [50]. There might also be some individual differences in the capacity to estimate appropriate portion sizes; for example, men tend to have more difficulties with this task [51]. Other studies have suggested that factors such as body mass index [52], the perceived healthiness of the product [53] or the energy density of the product [50] might play a role.

## Interventions Aimed at Portion Size

**Fig. 1:**
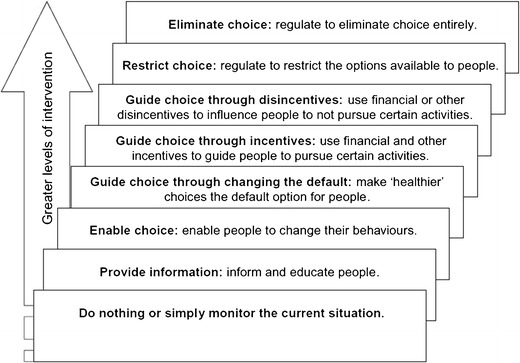
A ladder of interventions. From *Public health*: *ethical issues*, with kind permission from the Nuffield Council on Bioethics. Accessible at http://nuffieldbioethics.org/wp-content/uploads/2014/07/Public-health-ethical-issues.pdf, © Nuffield Council on Bioethics 2007

Although we know increasingly more about the portion-size effect and its working mechanisms, interventions tackling the portion-size effect are still scarce. Nevertheless, some interventions have been developed and tested recently (see Table 1). Interventions can be categorized according to the ladder of interventions of the British Nuffield Council on Bioethics [65], starting with simply providing information and ending with the more rigorous intervention of eliminating choice (i.e. not offering large portion sizes at all, see Fig. 1).

**Table 1:** Recent portion-size interventions

Step on ladder of intervention	Intervention	Recent evidence
Provide information	Labelling	Different types of labelling (reference size combined with calorie and GDA) showed no or at most a very small effect (Vermeer et al. [54]) A pictorial serving size recommendation may be an effective strategy in decreasing intake from larger packages (Versluis et al. [55])
	Naming and framing	Consumers respond to naming, independently of the actual size. Large sounding names (e.g. ‘double’) led to a decrease in intake (Just and Wansink [56])
	Website	Educational website was effective in increasing awareness of reference serving sizes and overeating triggers (Poelman et al. [31])
Enable choice	Provide segmentation cues in food packaging	A segmentation cue in the form of a red potato chip was effective in decreasing consumption (Geier [57])
	Provide a smaller portion alongside the larger one	Introducing a smaller sized hot meal in the worksite cafeteria alongside the larger sized meal led 10% of consumers to choose the smaller (Vermeer et al. [58]) Introducing a smaller sized entrée in a worksite cafeteria and at a commercial restaurant alongside the larger size led to a decrease in energy intake (Berkowitz et al. [59])
	Implementation intentions	Using implementation intentions (specific behavioural plans, using an if—then structure) led to a decrease in the number of sweets selected (van Koningsbruggen [60])
	Stop-signal training	Stop-signal training (a computer task, using images of high caloric, palatable foods versus non-food-related images, teaching individuals not to respond to the food images) led to a decrease in the number of sweets selected (van Koningsbruggen [60])
	Portion control strategies	Use of 32 portion control strategies was promoted in an educational programme. After 3 months, the programme was effective in decreasing BMI; the effects were mediated by portion control behaviour (Poelman et al. [31]; Poelman et al. [61•])
	Mindfulness exercise	Short mindful exercises were not effective in eliminating the portion-size effect (Cavanagh et al. [62]. The portion size effect on food intake. An anchoring and adjustment process?)
Guide choice through changing the default		–
Guide choice through incentives or disincentives	Remove the incentive to buy large amounts, which are seen to provide more value for money Provide more value for money when buying a smaller amount	Proportional pricing showed no effects in worksite cafeterias for a smaller and larger hot meal (300 vs. 500 g). Price differences were possibly too small due to lower prices in worksite cafeterias in general (Vermeer et al. [58]) –
Restrict choice	Limit sugar-sweetened beverages sold in restaurants to a maximum of 473 ml (restriction, as proposed in New York in 2012)	A modest reduction in calories is to be expected, especially among young adults and children who are overweight (Wang and Vine [63])
Eliminate choice	Decrease portion size	A reduction in the size of candies with 50% led to a decrease in energy intake among young adults (Marchiori et al. [64])

Ladder of intervention: British Nuffield Council on Bioethics, 2007

As Table 1 shows, most of the interventions that have been developed and tested are on the lower rungs of the ladder. Some interventions showed promising results, such as using segmentation cues in food packaging [57], forming implementation intentions [60] and the use of other self-regulatory, portion control strategies [31, 61•].

Studies with portion size as the key focus in weight loss interventions are still very rare. A study by Poelman et al. [17] showed that a decrease in BMI can be achieved by focusing on different strategies, solely to control food portion sizes. The effects on BMI were mediated through portion control behaviours [61•]. This is in accord with the findings of the SHED-IT trial, where portion size was found to be one of the mediators of the intervention effect on body weight at 6 months, alongside physical activity [66].

More research into interventions aimed at portion size, in which all rungs of the ladder are taken into account, is required. The higher rungs of the ladder might not be reached without government regulation and thus government action will need to be combined with non-regulatory interventions to ensure use of the entire ladder of interventions aimed at tackling the portion-size effect (see e.g. Marteau et al. [67]). Marteau et al. [67] also noted that public acceptance is of crucial importance if governments and other private parties are to act [67]; however, public acceptance of the government intervening in the food environment is in general rather low. In their study of the public acceptance of policies to reduce the consumption of sugar-sweetened beverages, Gollust et al. [68] found that American consumers appeared to be most in favour of labelling and restricting sales on school property, while the least support was found for taxes and portion-size restrictions. It could well be that public acceptance decreases as interventions climb the ladder of interventions although this might be slightly different in the case of interventions targeted at children [68]. More insight into the public acceptance of policy measures targeted at portion size will help in the development of strategies to enhance acceptance of policies.

In addition to public acceptance, it is also of importance to include intermediaries, such as restaurant owners in the development of strategies to control portion size. For example, Gase et al. [69] studied a voluntary programme in which restaurants offered reduced sized portions. Although they concluded that the programme was feasible, many barriers to offering reduced sized portions were identified by the restaurant owners, such as logistical barriers and concerns about revenue loss. Moreover, healthy eating was mainly seen as the responsibility of the consumer [69].

## Measurement Instruments

With the increasing attention being paid to interventions, the need for measurements of portion control behaviour has emerged. In recent years, three new instruments have been developed (see Table 2). Fast et al. [70] developed a portion control self-efficacy scale, which showed good validity and reliability, with scores on the scales associated with dieting success [70, 31]. derived 32 portion control strategies from the literature, with the overall Cronbach’s *α* of the scale valued at .82 and showed an association between the use of portion control strategies and reduced BMI [31].

**Table 2:** Measurement instruments portion control behaviour

Instrument and authors	Type of instrument
Portion control self-efficacy (Fast et al. [70])	12-item questionnaire on a 5-point scale, measuring self-efficacy toward portion control
Portion control strategies (Poelman et al. 2013)	32-item questionnaire on a 5-point scale, measuring strategies to control the amount eaten. Strategies concern: purchase behaviour; meal and package sizes; stockpiling; food exposure and unplanned eating; mindless eating; and dining out, all-you-can-eat and take away food
Portion control practices (Spence et al. [71])	15-item questionnaire on a 4-point scale, measuring the use of portion control practices. 3 sub-scales include measurement strategy scale, eating strategy scale and purchasing strategy scale

The portion control practices questionnaire developed by Spence et al. [71] consists of three subscales: the measurement strategy (use of guidance, i.e. by means of suggested serving sizes), the eating strategy (practices such as eating slowly) and the purchasing strategies (buying or ordering smaller amounts). Cronbach’s *α* of all subscales was ≥.78, and an association was found between the eating strategy scale and reduced BMI, whereas both the eating and purchasing strategies were negatively associated with pizza consumption [71]. These studies by Poelman et al. (2013) and Spence et al. [71] suggest that portion control strategies may improve food intake and support weight control. However, more research is needed into the validity of portion control behaviour scales and the associations with food intake and BMI [31, 71].

## Conclusion and Further Directions

Clearly, there is considerable evidence for the portion-size effect. However, there is still a lot of work to be done with respect to interventions targeting portion size. Although we conclude that a promising start has been made in relation to interventions targeting portion size, it is not yet clear what type of interventions work best, for whom and in what context [72]. Specific attention should be directed to the public acceptance of portion-size interventions and in particular portion-size policies. Based on the very limited number of studies thus far, targeting portion size seems a promising strategy for weight loss interventions but this should be explored further. Interventions should be developed and tested in real-life settings [18]. It is of importance to include the whole spectrum of potential interventions, from providing information to eliminating choices, from voluntary changes to regulatory interventions [54]. Moreover, valid measurements should be developed to evaluate the effects of interventions. While a few portion control scales have been developed, more research is needed into their validity and the associations with food intake as well as BMI.
